# A survey of midwifery graduates’ opinions about midwifery education in Iran: a cross-sectional study

**DOI:** 10.1186/s12909-021-02764-y

**Published:** 2021-06-10

**Authors:** Monireh Toosi, Maryam Modarres, Mitra Amini, Mehrnaz Geranmayeh

**Affiliations:** 1 Department of Reproductive Health and Midwifery, School of Nursing and Midwifery, Tehran University of Medical Sciences, Tehran, Iran; 2 Department of Reproductive Health and Midwifery, Member of Nursing and Midwifery Care Research Center, School of Nursing and Midwifery, Tehran University of Medical Sciences, Tehran, Iran; 3 Clinical Education Research Center, Shiraz University of Medical Sciences, Shiraz, Iran; 4 Department of Reproductive Health and Midwifery, School of Nursing and Midwifery, Tehran University of Medical Sciences, Tehran, 1419733171 Iran

**Keywords:** Education, Midwifery education, Clinical education, Student, Midwifery, Satisfaction

## Abstract

**Background:**

Attaining high-quality education requires continuous evaluation and revision of the curriculum. The view of the graduate students can provide valuable insight into the necessary evaluations and revisions. Therefore, this study aimed to evaluate the opinions of midwifery graduates about midwifery education in Iran

**Methods:**

This was a descriptive cross-sectional study and the research data were collected through a census sampling procedure from all (82) midwifery graduates of the Nursing and Midwifery School of Shiraz University of Medical Sciences between 2018 and 2020. The data collection instrument was a validated researcher-made questionnaire derived from the Graduation Questionnaire (GQ) developed by the Association of American Medical Colleges (AAMC). The data were then analyzed using SPSS 22.

**Results:**

In this study, about 62% of the graduates were satisfied with the quality of the midwifery education they had received during the four-year program. Moreover, 61% of the graduates maintained that theoretical courses were well-integrated with the clinical experience they needed. The quality of the internships in different wards and departments was also evaluated from the viewpoints of the graduates, and the results indicated that they were relatively satisfied with their internship experiences. However, only 40% of the graduates were satisfied with the quality of their clinical evaluation, since they faced the most significant challenges in the clinical and maternity wards (47%) with the midwifery staff and gynecology residents and found the quality of facilities in the clinical program to be lacking. According to the participants, the quality of teaching was not good for some courses such as biochemistry and microbiology.

**Conclusion:**

It seems that the midwifery curriculum needs to be constantly revised, aiming to improve student satisfaction with their midwifery education. Some effective measures in this regard are employing experienced professors, developing cooperation between midwifery instructors and clinical departments, and trying to improve the educational environment. Attention to the improvement of facilities and equipment and agreement between the content of the theoretical education and practical topics are also recommended to improve the quality of midwifery education.

## Background

Human resources constitute the cornerstone of the health system, and midwifery personnel as an integral part of human resources play a major role in promoting community health. Midwifery schools are responsible for training professional staff who can be effective in providing health services and shaping health policies. These educational centers are required to educate individuals with the adequate ability, knowledge, and skills to apply their learnings in practice and have adequate management skills for preventing and dealing with critical situations [1, 2] . Iran’s health system and the Ministry of Health have incessantly accentuated the key role of midwives in achieving national and international ideals for reproductive, maternal, neonatal, and infantile health. Given the importance of midwifery education in promoting community health, improving midwifery care, and reducing maternal and neonatal mortality, it is necessary to improve the quality of midwifery education, especially midwifery clinical education [3]. In Iran, midwifery students are admitted to a four-year university program via a national exam. The midwifery training program has been designed by the Ministry of Health and Medical Education in a single curriculum for all universities across the country. A significant part of the curriculum is devoted to the acquisition of clinical skills, during which students acquire skills in groups of four to eight in clinical settings. The students must participate in at least 60 natural deliveries and successfully pass a comprehensive midwifery exam in order to graduate. The clinical training course is an opportunity to acquire, practice, and develop clinical skills, during which students learn the necessary practical skills for professional midwifery activities [4]. However, the results of many studies in Iran have shown a relatively deep gap in the theoretical education process and clinical practice of midwifery students. Accordingly, the existing clinical education does not transfer the necessary ability to achieve appropriate clinical competence to students [5]. Evidence has also indicated that the professional skills of students have decreased compared to the previous decade [6, 7]. Accordingly, despite adequate theoretical knowledge, new graduates lack skills and efficiency in clinical settings [8]. In other words, clinical education has failed in its goals of training skilled people and improving the quality of care services [9]. In this context, various studies have demonstrated that the existence of multiple problems in clinical settings has prevented the achievement of educational goals [10]. Therefore, it seems that midwifery education needs fundamental changes [11]. In 2012, the standard program for the midwifery profession stated that the training provided in the midwifery curriculum should ensure that the students are prepared to practice the profession in accordance with the standards. According to these standards, midwifery students must have an acceptable level of ability to provide clinical services on patients’ bedsides, while the educational goals specified in the curriculum and the theoretical and practical training must ensure the provision of these capabilities. In addition, midwifery students must have the necessary self-efficacy to perform the assigned tasks [4]. The necessary skills have been established in different countries to train midwifery students. The International Confederation of Midwives published the minimum standards for clinical midwifery education in 2006 and then in 2008 [12]. Based on these standards, midwifery schools in Iran should educate graduates with the skills necessary for the disease prevention, treatment, and health promotion of mothers and infants. To have maximum efficiency, midwifery students should participate in theoretical classes while also acquiring clinical skills through practice and gaining experience in clinical settings [13]. The midwifery clinical education in Iran is challenged by certain factors. Due to the shortage of clinical professors with sufficient knowledge and skills for teaching as well as practical and specialized practice, educational managers have tasked clinical personnel with teaching midwifery students through traditional midwifery education methods [14]. Although most clinical preceptors possess useful clinical experience, when it comes to education, those who adopt the role of clinical teachers should be up to date on the latest scientific information to teach theoretical topics. Still, due to their departure from academic education or because of time restrictions, some clinical personnel change the process of care, eliminate many standard care steps, and create so-called shortcuts in the accurate implementation of care [15]. However, to meet educational standards, these care practices should be performed in their complete and up-to-date form by the teacher. In the traditional midwifery education model, most students experience educational contrasts and confusions; on the one hand, theoretical topics are transferred to them in their academic form, and on the other hand, they encounter teachers in clinical settings whose performance runs contrary to those theoretical topics. In Iran, clinical settings such as hospitals are separate from academic settings, and midwifery professors are in fact guests in clinical settings and rarely wield administrative power [14]. These conditions cause conflicts between midwifery professors and the personnel, doctors, and OB/GYNs. To resolve these problems and integrate universities and clinical settings, measures such as the implementation of the Midwifery Clinical Faculty Model (MCFM) have been taken in Iran. In 2014, the clinical education revival project and the deployment of midwifery faculty members at hospitals and clinical settings were notified by the Iranian Ministry of Health and Medical Education (MoHME) to the universities across the country [16]. To promote the students’ clinical skills and general capabilities, this educational model was piloted in some Iranian universities of medical sciences, including the Shiraz University of Medical Sciences, with the deployment of midwifery faculty members on three shifts (morning, afternoon, night). In the Midwifery Clinical Faculty Program, students are required to provide low-risk mothers with the required care during labor and delivery and to assist in the delivery of high-risk groups under the close supervision of clinical instructors. Students are also responsible for the continuation of maternal and neonatal care in the fourth stage of labor. The Midwifery Clinical Faculty Program, as a competency-based education model, has tried to use new training methods (focus group discussion, problem-solving, etc.) that can help students to achieve optimal performance in clinical environments. Evaluation is an essential part of academic education, and the result of principled evaluation can be a basis for reforming and revising higher education curricula in the country, which can ultimately improve the academic levels of universities [17]. One of the most appropriate approaches to address educational problems in midwifery departments is to evaluate and determine the satisfaction of the graduates of these disciplines as healthcare providers across the country [18–20]. Given the importance of promoting midwifery education, especially clinical education, the researchers decided to design and implement the present study to assess the determine the opinions of midwifery graduates about midwifery education in Iran.

This study has two specific objectives:

- Assessing the midwifery education and curriculum.
- Assessing the midwifery clinical education with an emphasis on the Midwifery Clinical Faculty Program.

## Methods

### Ethical statement

The present study was approved by the Ethics Committee of Tehran University of Medical Sciences (IR.TUMS.FNM.REC.1398.057). After introducing herself to the participants, the researcher explained the study objectives and assured them about the confidentiality of their information and their authority to reject the invitation or participate/withdraw from the study. She also requested the participants to sign the written informed consent forms.

### Study design

This descriptive cross-sectional study included all midwifery program alumnae (82) who were trained through the midwifery clinical faculty program from 2017 to 2019 and were graduated from the Nursing and Midwifery School of Shiraz University of Medical Sciences.

### Setting and context

Despite the efforts made in recent decades to promote midwifery education in Iran, research shows little success in achieving the goals envisioned for midwifery education and reports inadequacies in graduates’ capabilities. These problems necessitate effective measures to boost the quality of midwifery clinical education [21]. Previous studies have shown that midwifery students were not satisfied with their clinical skills, supervision, and access to information before clinical education [22, 23]. In Iran, midwifery students are admitted to a four-year university program via a national exam, and only female students are allowed to study midwifery.

From the third semester, their clinical learning is begun under the supervision of clinical instructors or clinical preceptors in clinical settings [12]. The standard method of clinical education in Iran is teacher-centered, and in some cases, the clinical personnel train midwifery students using traditional midwifery education methods. This method may not convey appropriate clinical experiences to students [14, 22]. The traditional midwifery clinical education model included group education with the periodical presence of professors in clinical settings and training provided by the maternity ward personnel. However, in the midwifery clinical faculty model (MCFM), students receive clerkship-based, continuous clinical education in the presence of midwifery professors with one-to-one interaction between the students and professors. Also, when the students clinically practice midwifery and childbirth for the first time, a midwifery teacher continuously explains different stages of childbirth to them [16].

### Subjects and sampling

Due to the limited statistical population, all 82 midwifery graduates of Shiraz University of Medical Sciences from 2017 to 2019 were selected as the research sample through the census method. Sampling started in January 2020 and lasted for ten months until October 2020.

The inclusion criteria included undergraduate midwifery students who have completed the clinical course during the Midwifery Clinical Faculty Program and graduated from Shiraz University of Medical Sciences. The exclusion criteria included graduates who have not received clinical training in the clinical faculty program. Finally, all 80 graduates passed the inclusion/exclusion criteria, and their data was used in statistical analysis.

First, permission from the faculty management was obtained. Next, participants received information about the study and their rights (e.g., the study’s aims, voluntary participation, confidentiality, anonymity, and right to withdraw from the study). Then, participants who had informed consent for the study completed a printed version of anonymous questionnaires distributed by the researchers. Finally, the data were analyzed (response rate: 100%).

### Research instruments

The data collection instrument was a researcher-made questionnaire designed based on a Graduate Questionnaire (GQ) developed by the Association of American Medical Colleges. Approved by the medical education and language specialists of Shiraz University of Medical Sciences, the questionnaire was translated for the first time into Persian by the experts in the Center for Medical Development, and Studies in Shiraz University of Medical Sciences validity and reliability were confirmed [23]. After making the necessary changes to the questionnaire and coordinating its items with the midwifery curriculum, the questionnaire was given to 12 midwifery faculty members, and their comments and suggestions were incorporated into the final version. Accordingly, the face validity and content validity of the questionnaire were confirmed. Moreover, the reliability of the questionnaire was confirmed using the internal consistency method. The internal consistency between the questionnaire items completed by 24 graduates was also confirmed by Cronbach’s alpha (α =0.942). The questionnaire consisted of eight sections as follows: 1) personal characteristics, 2) quality of midwifery education, including quality of courses and internship, 3) quality of other instructions, including clinical decision-making, clinical care, evidence-based midwifery, and community-based midwifery, 4) student services, 5) overall attitude towards the quality of the Clinical Faculty Curriculum, 6) future career plans, 7) general problems of the college years, and 8) midwifery school experiences. Their responses were given on a five-point Likert scale from 1 (poor) to 5 (very good). The questionnaire was administered after obtaining ethical clearance from the authorities (code number: IR.TUMS.FNM.REC.1398.057) and written informed consent from the participants.

### Statistical analysis

Next, the data were entered into SPSS 22 and were analyzed using descriptive statistics (mean, frequency). Frequency and percentage were used for describing categorical variables (i.e., age, marital status, year of graduation, and employment). The Kolmogorov-Smirnov test was used to verify the normality of the data. One-way analysis of variance (ANOVA) tests was employed to determine significant variations in their scores in the final clinical exam. All statistical tests were carried out at a 95% confidence level using SPSS 22 and the significance threshold was set at 0.05. (SPSS Inc., Chicago, IL, USA).

## Results

This study was conducted on 82 participants (100% female) who graduated from the undergraduate midwifery program at Shiraz University of Medical Sciences between 2017 and 2019 (response rate: 100%). The mean age of the participants was 25 ± 5.9 years (Table 1).

**Table 1:** Individual and educational characteristics of the midwifery graduates

Variable	Category	N (%)
Age (years)	25>	53 (64.6)
	25–35	20 (24.4)
	35<	9 (11)
Year of Graduation	August 2018	24 (29.3)
	September 2019	24 (29.3)
	June 2020	18 (22)
	Feburary2020	16 (19.4)
Marital status	Single	44 (53.7)
	Married	38 (46.3)
Graduate grade point average	14>	3 (3.7)
	14–18	78 (95.1)
	18<	1 (1.2)
Employment of graduates	Unemployed	47 (57.3)
	Employment in the midwifery profession	26 (31.7)
	Employment in a non-midwifery profession	9 (11)

A. The acceptable minimum grade point for each lesson is 10 (range 0 to 20), and the average grade point average for the course is 12. Also, after passing all theoretical and clinical courses, the student must pass the final clinical exam and acquire a minimum score of 12 (Source: Midwifery Undergraduate Curriculum, Iranian Supreme Medical Sciences Planning Council, Available at http://mbs.behdasht.gov.ir/index.aspx?siteid=176&fkeyid=&siteid=113&pageid=18442)

Based on the evaluation tool, six indicators related to curriculum goals, curriculum content, curriculum tests, clinical practice, readiness to enter internship, and quality of midwifery education were evaluated from the perspective of the graduates (Table 2).

**Table 2:** Participant viewpoints on the midwifery curriculum

Statement	Strongly disagree	Disagree	Neutral	Agree	Strongly agree
1. The aims stated in the midwifery curriculum are clear to students.	2 (2.4%)	15 (18.3%)	18 (22%)	43 (52.4%)	4 (4.9%)
2. The contents of the required courses are well integrated into the midwifery curriculum.	11 (13.4%)	19 (23.2%)	9 (11%)	37 (45.1%)	6 (7.3%)
3. The types of exams are closely related to the educational objectives.	6 (7.3%)	15 (18.3%)	23 (28%)	29 (35.4%)	9 (11%)
4. Course contents include appropriate and relevant clinical examples.	10 (12.2%)	16 (19.5%)	17 (20.7%)	35 (42.7%)	4 (4.9%)
5. The content of theoretical courses prepares the students for the internship.	10 (12.2%)	17 (20.7%)	10 (12.2%)	33 (40.3%)	12 (14.6%)
6. The quality of midwifery education is generally satisfactory	11 (13.4%)	10 (12.2%)	10 (12.2%)	44 (53.7%)	7 (8.7%)

The findings showed that 57.3% (Agree and strongly agree) of the graduates claimed that the midwifery curriculum is clear to students. About 52.4% of the graduates agreed (Agree and strongly agree) to integrate midwifery content into the midwifery curriculum. Overall, 62.4% of the graduates were satisfied with the quality of the midwifery education over the four years.

Regarding the opinion of the graduates about each of the midwifery courses, the highest percentage of answers to the excellent option was related to the pregnancy course 1 (Natural Pregnancy) in 41.5% of cases, and the lowest percentage of the excellent option was related to the biochemistry course with 6.1% (Table 3).

**Table 3:** Participant viewpoints about the quality of undergraduate midwifery courses

The offered courses	Poor N (%)	Fair N (%)	Good N (%)	Excellent N (%)	Not applicable N (%)
1. Quality of education in Biochemistry course	20 (24.4%)	25 (30.5%)	21 (25.6%)	5 (6.1%)	11 (13.4%)
2. Quality of education in Genetics course	14 (17.1%)	27 (32.9%)	25 (30.4%)	8 (9.8%)	8 (9.8%)
3. Quality of education in Family Planning course	18 (22%)	21 (25.6%)	27 (32.9%)	9 (11%)	7 (8.5%)
4. Quality of education in the Anatomy course	4 (4.9%)	18 (22%)	42 (51.2%)	17 (20.7%)	1 (1.2%)
5. Quality of education in Neonates course	6 (7.3%)	21 (25.6%)	34 (41.5%)	20 (24.4%)	1 (1.2%)
6. Quality of education in Pregnancy and Childbirth 4 course (Internal Medicine and Surgery in Pregnancy and Childbirth)	3 (3.6%)	13 (15.9%)	35 (42.7%)	30 (36.6%)	1 (1.2%)
7. Quality of education in Principles and Techniques of Nursing and Midwifery, and Procedures in the Operating Room and Childbirth course	3 (3.6%)	13 (15.9%)	35 (42.7%)	26 (31.7%)	5 (6.1%)
8. Quality of education in Pregnancy and Childbirth 1 course (Natural Pregnancy)	3 (3.6%)	7 (8.5%)	34 (41.5%)	34 (41.5%)	4 (4.9%)
9. Quality of education in Microbiology course	20 (24.4%)	24 (29.3%)	23 (28%)	9 (11%)	6 (7.3%)
10. Quality of education in the Pregnancy and childbirth 2 course (Natural Childbirth, Safe Physiologic Childbirth, and Labor Pain Relief Methods)	2 (2.4%)	16 (19.5%)	31 (37.8%)	32 (39%)	1 (1.2%)
11.Quality of education in Pharmacology	19 (23.2%)	17 (20.7%)	27 (32.9%)	16 (19.5%)	3 (3.7%)
12. Quality of education in Gynecological Diseases and Infertility course	4 (4.9%)	13 (15.9%)	37 (45.1%)	28 (34.1%)	0 (0.0%)
13. Quality of education in Physiopathology and Internal Medicine course	9 (11%)	29 (35.4%)	25 (30.5%)	18 (21.9%)	1 (1.2%)
14. Quality of education in Pregnancy and Childbirth 3 course (Pregnancy and Non-natural Childbirth)	2 (2.4%)	10 (12.2%)	41 (50%)	29 (35.4%)	0 (0.0%)

According to the graduates, the excellent clinical experience belonged to childbirth internship (45.1%) and gynecological diseases internship (32.9%). However, the graduates believed that radiology internship (11%) and surgery internship (12.2%) had the lowest quality (Table 4).

**Table 4:** Participant viewpoints about the quality of their experience with clinical internship

Quality of the clinical experience acquired in clinical internships	Poor N (%)	Fair N (%)	Good N (%)	Excellent N (%)	Not applicable N (%)
1-Ranking of the quality of the clinical experience in internal medicine	7 (8.5%)	31 (37.8%)	33 (40.3%)	11 (13.4%)	0 (0.0%)
2.Ranking of the quality of clinical experience in childbirth	1 (1.2%)	9 (11%)	35 (42.7%)	37 (45.1%)	0 (0.0%)
3.Ranking of the quality of clinical experience in pediatrics	9 (11%)	24 (29.3%)	33 (40.2%)	14 (17.1%)	2 (2.4%)
4.Ranking of the quality of clinical experience in neonates	6 (7.3%)	20 (24.4%)	33 (40.3%)	23 (28%)	0 (0.0%)
5. Ranking of the quality of clinical experience in radiology, ultrasound, and electro logy in midwifery	27 (32.9%)	22 (26.8%)	18 (22%)	9 (11%)	6 (7.3%)
6. Ranking of the quality of clinical experience in surgery	17 (20.7%)	32 (39%)	22 (26.9%)	10 (12.2%)	1 (1.2%)
7.Ranking of the quality of clinical experience in gynecological diseases and infertility	5 (6.1%)	16 (19.5%)	33 (40.3%)	27 (32.9%)	1 (1.2%)
8.Ranking of the quality of internship in domains of fertility, mother and child health, and family planning	11 (13.4%)	14 (17.1%)	34 (41.5%)	23 (28%)	0 (0.0%)
9.Ranking of the quality of principles of management and its application in midwifery	13 (15.9%)	20 (24.4%)	23 (28.0%)	14 (17.1%)	12 (14.6%)

Moreover, 45.1% of the students were satisfied (agree and strongly agree) with student support services of student vice-presidency, 53.7% were satisfied with access to the administration and teaching affairs office of the midwifery school, 43.9% were satisfied with student support and financial services, 37.8% were satisfied with student welfare services, 40.2% were satisfied with student health and insurance services, and 74.4% were satisfied with access to library services (Table 5).

**Table 5:** Participant viewpoints about the quality of services and student affairs

Statement	Strongly disagree	Disagree	Neutral	Agree	Strongly agree
1. Satisfaction with the cooperation of the student vice-presidency	5 (6.1%)	10 (12.2%)	30 (36.6%)	30 (36.6%)	7 (8.5%)
2. Satisfaction with the performance of the school’s administration and teaching affairs office with students	2 (2.4%)	12 (14.6%)	24 (29.3%)	31 (37.8%)	13 (15.9%)
3. Satisfaction with counseling-supportive and financial services	7 (8.5%)	14 (17.1%)	25 (30.5%)	24 (29.3%)	12 (14.6%)
4. Satisfaction with student welfare services	14 (17.1%)	21 (25.6%)	16 (19.5%)	23 (28.0%)	8 (9.8%)
5. Satisfaction with health and insurance services	10 (12.2%)	19 (23.2%)	20 (24.4%)	23 (28.0%)	10 (12.2%)
6. Satisfaction with library services	4 (4.9%)	5 (6.1%)	12 (14.6%)	17 (20.7%)	44 (53.7%)
7. Satisfaction with the computer center and computer services	10 (12.2%)	7 (8.5%)	10 (12.2%)	14 (17.1%)	41 (50%)
8. Satisfaction with the Student study space	9 (11%)	5 (6.1%)	11 (13.4%)	13 (15.9%)	44 (53.6%)
9.Satisfaction with Student rest space	42 (51.2%)	7 (8.5%)	8 (9.8%)	5 (6.1%)	20 (24.4%)

B. The satisfaction level was measured through the sum of “agree” and “strongly agree”

Overall, the quality of the Clinical Faculty Curriculum (14.6% Excellent) and the quality of welfare facilities in the Midwifery Clinical Faculty program (4.9% Excellent) were the lowest indicators in the evaluation of the quality of the Clinical Faculty Curriculum from the viewpoints of the midwifery students (Table 6).

**Table 6:** Participant viewpoints about the quality of the Midwifery Clinical Faculty Program

The quality of the Midwifery Clinical Faculty program	Excellent N (%)	Good N (%)	ModerateN (%)	Poor N (%)	Very poor N (%)
1. What is the quality of academic topics offered in the clinical faculty curriculum?	20 (24.4%)	42 (51.2%)	20 (24.4%)	0 (0.0%)	0 (0.0%)
2. What is the quality of the professors teaching in the clinical faculty curriculum?	15 (18.3%)	34 (41.5%)	31 (37.8%)	1 (1.2%)	1 (1.2%)
3. What is the quality of clinical skills taught in the clinical faculty curriculum?	14 (17.1%)	33 (40.2%)	30 (36.6%)	4 (4.9%)	1 (1.2%)
4. What is the quality of educational facilities and equipment available in the clinical faculty curriculum?	6 (7.3%)	18 (22%)	40 (48.8%)	15 (18.3%)	3 (3.6%)
5. What is the quality of time planning in the clinical faculty curriculum?	13 (15.9%)	26 (31.7%)	33 (40.2%)	9 (11%)	1 (1.2%)
6. What is the quality and quantity of welfare facilities in the clinical faculty curriculum?	4 (4.9%)	13 (15.8%)	31 (37.8%)	24 (29.3%)	10 (12.2%)
7. What is the quality and quantity of computer facilities and Internet access in the clinical faculty curriculum?	5 (6.1%)	19 (23.2%)	27 (32.9%)	20 (24.4%)	11 (13.4%)
8. What is the quality and quantity of academic resources and bases in the clinical faculty curriculum?	9 (11%)	22 (26.8%)	32 (39%)	16 (19.5%)	3 (3.7%)
9. What score do you give to the overall quality of the clinical faculty curriculum?	12 (14.6%)	35 (42.7%)	30 (36.6%)	4 (4.9%)	1 (1.2%)

This study showed significant difference between the final exam scores of midwifery clinical faculty program graduates (2018–2020) and those of graduates who did not participate in the midwifery clinical faculty program (September 2017(.The results of the LSD test showed a significant difference between the final exam scores of the control group (September 2017(and the other groups (2018–2020) participating in the program. Participation in the midwifery clinical faculty program improved graduates’ final exam results (Table 7).

**Table 7:** Comparison of the average results of the final clinical exam

Variable	Midwifery graduates	Mean ± SD	Sum of Squares	df	Mean Square	F	Sig.
Score of final clinical exam	Graduate (September2017)	14.59 ± .1.04	16.668	4	4.167	4.548	0.002
	Graduate (August 2018)	15.33 ± .0.74					
	Graduate (September2019)	15.25 ± .1.06					
	Graduate (June2020)	15.80 ± .0.77					
	Graduate (Feburary2020)	15.24 ± .1.06					

According to the findings of this study, the participants had the largest problems in the clinical and maternity wards (47%) with nursing staff and gynecology residents. In response to the question about retaking midwifery as a field of study, about 48% of the participants answered negatively, 37% said that they would opt again for midwifery, and about 15% did not have a particular opinion. This level of dissatisfaction is noteworthy and requires further investigation. Among the participants, 79% were satisfied with the teaching of clinical decision-making and clinical care skills, such as patient interviewing skills, patient examination, diagnosis of diseases, inpatient care, and communication with patients and physicians, and they found these instructions effective. Additionally, 72% of the graduates were satisfied with midwifery education in specific areas, such as midwifery management, healthcare improvement, midwifery data recording, confidentiality, privacy, midwifery economics, and midwifery law. Moreover, 58% of the participants were satisfied with evidence-based midwifery education, such as interpretation of clinical data, interpretation of test results, and decision analysis, and 73% of the graduates evaluated the skill they gained for teamwork with other medical teams as satisfactory. Besides, 89% of the participants acknowledged that they had acquired satisfactory levels of skills in confidentiality, maintaining the patients’ privacy, and observing the midwifery ethics during their internship. In terms of testing and assessment, 96% of the internships were evaluated by clinical professors during the internship. In this regard, 93% of the students had oral exams, while 76% had written exams. Along these lines, 90% of the participants were familiar with logbooks and were evaluated based on their internship logbooks. However, only 40% of the graduates were satisfied with the quality of their clinical evaluation. The findings showed that 61% of the graduates claimed that the topics of theoretical courses were well-integrated with the clinical experience they needed. The quality of the internship in different wards was also evaluated from the graduates’ viewpoints, and the results revealed their moderate satisfaction with all clinical wards. In addition, there was no statistically significant difference in terms of satisfaction and other items of the questionnaire between different groups of midwifery graduates between 2018 and 2020.

## Discussion

Knowing graduates’ views on the appropriateness of the goals of the curriculum and the way they are implemented in midwifery schools can provide educational planners and policymakers with valid information. The results of the evaluation of the alumnae’s views demonstrated that 62% of the graduates were satisfied with the quality of the midwifery education they had received over the four-year program. In a study on the relationship between communication skills and course satisfaction among midwifery students, Etebari asl et al. found that most midwifery students were engrossed in their field of study and were relatively satisfied with the quality of their curriculum [24]. Jamilian et al. found that the midwifery students were relatively satisfied with the educational quality of their curricula and the mean score of satisfaction with the field of study was moderate among the students [24]. Vanaki reported that students in Ahvaz were discontent with the educational quality of their curriculum [25]. The discrepancy among these results might be due to the influence of different environmental circumstances on students’ perceptions. The results of the present study showed that the graduates evaluated the quality of midwifery education in theoretical and practical courses as relatively satisfactory. Moreover, the quality of students’ clinical skills education was found to be satisfactory in the clinical faculty program. Yet, further measures are required to achieve excellence in the quantity and quality of services. Clinical learning is the main part of the midwifery curriculum [26]. A key strategy for ameliorating clinical education is to use capable instructors both in theoretical and clinical courses. The present study results also showed that 60% of the graduates assessed the presence and teaching of professors in the Clinical Faculty Curriculum as appropriate. One of the most critical expectations of students in effective clinical education is the availability of midwifery instructors and their adequate communication and interaction with the students [27]. Adequate relationship with students is effective in motivating them and raising their interest in clinical education. It is one of the most important agents for inhibiting stress in midwifery students and the stressful delivery room environment [28]. Such circumstances can pave the way for better learning and will allow for greater coordination between the instructors and students [29]. Such circumstances can pave the way for better learning and will allow for greater coordination between the instructors and students [29, 30]. Similarly, Dehghani et al. found that appropriate professional relationships could create a positive environment for trainees. Additionally, the involvement of professors and clinical staff in student support and training was identified as contributing to promoting clinical education and enhancing student confidence [28]. Iranian midwifery students have a slim chance of acquiring independent experiences in clinical learning. This condition leads to unpleasant fears and stress [21]. So, the support provided by instructors and clinical staff is essential for positive learning experiences [26]. Midwifery students are greatly influenced by their professors’ teaching methods and expectations. Experts believe that clinical educators have a significant effect on enhancing the quality of clinical education and can make clinical experiences enjoyable for students [31]. Being publicly criticized by their clinical instructor causes fear and increases the stress levels of the students. Iranian students sometimes face such issues, which remain unnoticed by the instructors [21, 31]. A practical clinical instructor should create appropriate communication and a supportive emotional climate to create a favorable environment for learning [32, 33]. Experienced instructors know how to communicate effectively with students and choose the right time and place for criticism and recommendations. Otherwise, these can damage students’ self-confidence and personality [33, 34]. Proper interaction and collaboration among clinical professors, staff, and students can play an important role in creating an appropriate clinical environment conducive to high-quality training of students [35, 36]. The gap between theory and practice and the clinical staff’s negative view of clinical instructors may also be among the causes of ineffective clinical education. Lukasse et al. showed that many clinical staff have a negative view of midwifery instructors who have not been in clinical practice for many years or are not updated. Old and outdated teaching methods lead to a gap between theory and practice [37]. Although one goal of the Midwifery Clinical Faculty Program was to boost the relationship between the educational and clinical environments, the alumnae believed that other clinical staff had poor cooperation with the students. Most of the problems encountered by the students in the clinical and maternity wards (47%) were related to the clinical staff and gynecology residents, indicating a deep gap between clinical and educational staff. A study in Ghana showed that environmental factors and interpersonal and academic relationships could negatively affect midwifery students, with the interpersonal stressors being the most potent [37]. Midwifery students experience certain problems during their academic and clinical education that may lead to uncertainty, dissatisfaction, and failure to adapt to their profession [38]. Most of these problems are driven by a wide range of potential issues in the clinical learning environment and interpersonal relationships that can affect students’ learning. While midwifery students usually have vast knowledge, they lack sufficient clinical skills, and they are unsuccessful in applying their theoretical knowledge in a stressful environment [39, 40]. The results of Ahmadi’s study showed that staff attitude toward midwifery students was evaluated at a moderate level and the students believed that the medical staff in the wards did not cooperate well in clinical settings [41]. Mutual interaction with other medical groups, common understanding, and shared objectives are essential for creating positive experiences for students in a learning environment [42]. The majority of midwifery graduates in the current study acknowledged that although their professors had empowered them regarding teamwork with medical workers from other disciplines in emergencies and to adhere to the principles of midwifery ethics in these special situations, midwifery staff and gynecology residents created many problems for them in the clinical and maternity wards. This indicated that the ward staff did not treat the students well and that they were not actively involved in training the students. In such an atmosphere, the students cannot communicate properly with the staff and often suffer from tensions. Since one of the goals of the clinical faculty curriculum is to reduce the gap between educational and clinical settings, it is necessary to put more effort into achieving this goal. A study conducted at Golestan University of Medical Sciences on the stressful experiences of midwifery students during clinical education in the labor room showed that the characteristics of the individuals who worked in the clinical wards and the educational environment where students spent their clinical experience had a great impact on their stress levels and the quality of their internship [29]. A study in Iran showed high levels of stress in midwifery students in clinical settings, with the stressors associated with the instructors causing more stress [43]. Another study seeking students’ evaluation of certain factors in the clinical learning environment at a Slovakian university revealed that a positive attitude and an appropriate collaborative atmosphere improved the students’ learning [44]. Inappropriate behaviors of ward staff with students, medical group interventions including gynecology residents, and lack of amenities in clinical departments were among the challenging factors in acquiring clinical skills in the present study, some of which were also reported by Rezaei [41]. To achieve the goals of the clinical faculty curriculum and to succeed in implementing this curriculum, efforts should be made to resolve this problem with other medical departments and to stabilize the position of midwifery instructors and students in the clinic. Elo’s study on students’ experiences in the clinical setting revealed that poor communication between staff and students could lead to the students’ disinterest in learning and their negative attitudes [45]. Ghafourifard also referred to barriers against the achievement of clinical goals, including lack of consistency between theoretical knowledge and clinical skills, vague internship goals, stressful clinical environment, reluctance of experienced professors to attend the clinic, lack of realistic evaluation, and lack of educational facilities [46]. In the current study, 65% of the graduates believed that they were ready to start their compulsory medical service program or enter the job market. This was consistent with the results of the research performed by Olga on students’ perceptions of their professional competencies in a Spanish university [46]. In contrast to the present study’s results, indicating that the graduates were prepared to enter the compulsory service program, Mousavi et al. reported that midwifery students believed they were poorly and moderately ready to enter the clinic and begin clinical practice [27]. The difference between these two findings could be attributed to the differences in the educational and clinical settings of the two studies. The presence of empowered faculty in the clinical faculty curriculum provides the opportunity to empower students and prepare them for entering the job market. In the present study, 73% of the graduates evaluated the library services of the midwifery school as appropriate. Furthermore, the availability of academic library resources and appropriate educational facilities of the school were referred to as one of the most important elements affecting the quality of education. Of course, an assessment of the viewpoints of the midwifery graduates in Shiraz indicated that the educational facilities of the faculty itself were more satisfactory in comparison to the educational conditions of the clinical centers. Graduates in Qazvin also evaluated the educational facilities of the school higher than those in clinical centers [32]. Another study conducted by Mousavi on clinical education problems in midwifery showed that the participants considered environmental factors in clinical settings as obstacles to achieving clinical education goals [27]. Ahmadnia and Salehabadi also disclosed that environmental factors, such as the great number of midwifery students in internships and the shortage of equipment, were barriers to clinical education [8, 47]. Evaluation of the Clinical Faculty Curriculum graduates in the current study indicated that the welfare and educational conditions and facilities of the clinical settings were not quite satisfactory. This finding highlighted the necessity to pay more attention to the improvement of clinical environments for the promotion of students’ learning. The results also showed that although 96% of clinical internships were evaluated by clinical professors, the evaluation system was not adequately satisfactory, and the majority of midwifery students were dissatisfied with the evaluation procedure and lack of ongoing feedback to students as well as non-standard and subjective evaluation procedures. Rezaei also reported dissatisfaction with the evaluation of internships and clinical educational environments and staff’s behaviors towards students [41]. In a study by Ahmadnia et al., students referred to the disconformity between evaluation forms and the existing conditions as well as different methods of evaluation by the instructors as educational problems and pointed to poor clinical evaluation procedures [47]. In the same line, Graham reported that non-standard evaluation was one of the most common barriers to clinical education from the students’ perspective [36]. In another study, Helminen found that the assessment process of students’ clinical practice lacked consistency. Great variety in the quality of assessment and differences in the perceptions of the mentors of the assessment forms were among the student assessment challenges [48]. Given the importance of the Clinical Faculty Curriculum in improving students’ educational conditions, it is necessary to review and change the students’ clinical assessment system in this curriculum.

## Conclusion

The current study results showed that 62% of the graduates were satisfied with the quality of the midwifery education they had received over the four-year program. Presenting some courses such as obstetrics and gynecology is desirable for students, but some courses such as biochemistry and microbiology need to be further reviewed and revised. Moreover, 61% of the graduates supported the integration of theoretical courses with clinical topics. Also, the level of student services from the students’ point of view in some cases, such as access to the vice chancellor for education and support and counseling services, has been at a moderate level. In contrast, welfare services have failed to reach a desirable level. This study clarifies some aspects of the curriculum to be further developed by the faculty to prepare midwifery graduates for clinical work. The results of this study showed that although the Clinical Faculty Curriculum at Shiraz University of Medical Sciences was in its incipient stage, the graduates were relatively satisfied with the quality of education. However, there were some deficiencies requiring revision at the discretion of educational planners. The effective implementation of the Clinical Faculty Curriculum requires the elimination of deficiencies such as poor communication between the staff of other medical departments and students in the clinic, poor quality of educational and clinical facilities, and poor quality of the students’ clinical assessment system. Since high-quality curricula can lead to the development of competent and capable students in the field of healthcare services, it is necessary to constantly evaluate and monitor the educational programs and review midwifery curricula with an emphasis on the elimination of weaknesses. The results of this study can act as a guide for midwifery education professionals in the revision of the curriculum of other universities. These can also help educators to design strategies for more effective clinical teaching. Given the complexity of the field of midwifery, using modern clinical training methods, such as the clinical faculty program, may enhance the efficiency of clinical training in Iran. It is recommended that school managers assess the students’ learning during clinical education, employ experienced instructors, and improve educational facilities and equipment. Future studies are recommended to evaluate concurrently the views of students, instructors, midwives, and school managers using a larger sample.

### Limitations

Due to the limited resources, time, location, and wide distribution of midwifery graduates, this study could not cover large groups of graduates. The small sample (limited to one university) and the cross-sectional survey design are the limitations of this study, affecting its generalizability. However, to our knowledge, this is the first study to evaluate the opinions of the midwifery graduates about midwifery education and the Midwifery Clinical Faculty Program in Iran.

## Data Availability

Data would be available by contacting the corresponding author.

## Abbreviation

MCFM: Clinical Faculty program
